# Ultrastructure of *Serratia liquefaciens* Grown at 7 mbar Under Simulated Martian Conditions

**DOI:** 10.3390/microorganisms13112466

**Published:** 2025-10-29

**Authors:** Andrew C. Schuerger, Karen L. Kelley

**Affiliations:** 1 Department of Plant Pathology, University of Florida, Gainesville, FL 32611, USA; 2 Electron Microscopy Core, Interdisciplinary Center for Biotechnology Research, University of Florida, Gainesville, FL 32611, USA; vau@ufl.edu

**Keywords:** Mars astrobiology, planetary protection, outer membrane vesicles, hypopiezotolerant bacteria, spacecraft microbiology

## Abstract

Cells of *Serratia liquefaciens* were grown on trypticase soy agar (TSA) for 28 d under Martian conditions of 7 mbar, 0 °C, and CO_2_-enriched anoxic atmospheres (called Mars low-PTA conditions). Earth controls were maintained for 24 h at 1013 mbar, 30 °C, and a standard pN_2_/pO_2_ gas composition. Cells were harvested at either 24 h or 28 d from TSA surfaces and processed for SEM and TEM imaging. Cells of *S. liquefaciens* grown under Earth conditions were uniform in shape and size, averaging approximately 1.25 µm in length and 0.5 µm in width. Fimbriae were observed on 10–20% of cells grown under Earth conditions. Key features of low-PTA grown cultures were (1) cells exhibited swollen blunt ends at sites of cell division tapering to unusually constricted points on the distal ends of progeny cells, (2) cell division appeared disrupted with division planes occurring at odd angles often forming right-angle oriented daughter cells, (3) some cells failed to form divisional planes resulting in long spiral and oddly shaped cells measuring up to 6–8 µm in length, and (4) fimbriae were lacking. Cell walls were found to be approx. 17% thinner when cells were grown in low-PTA environments compared to lab-standard conditions.

## 1. Introduction

The effects of low pressure (def. here as ≤25 mbar) on microbial metabolism, growth, and evolution of terrestrial microorganisms are directly relevant to predicting the forward contamination of Mars, developing a search strategy for life on the Martian surface or shallow subsurface, and studying the active aerobiology in Earth’s stratosphere. Several low-pressure-tolerant bacteria (called hypopiezotolerant bacteria) capable of metabolism and growth at 7 to 25 mbar have been described [1,2,3,4,5,6,7], including the Gram-negative bacterium, *Serratia liquefaciens*. In addition, the methanogenic archaeon *Methanosarcina barkeri* has been shown to be metabolically active and to produce methane at 7–12 mbar [8]. In contrast, fungi do not appear capable of growth at pressures similar to the surface of Mars (approx. 7–10 mbar [4]). Although several lichen and cyanobacteria species have survived exposures to Mars-like low-pressure conditions between 7 and 10 mbar [9,10,11,12], it has not yet been demonstrated that lichens or cyanobacteria are capable of active growth and cellular replication under Mars-like surface conditions between 7 and 10 mbar.

Several studies have explored the effects of low pressures on the growth [3] and metabolism [5,6,7] of *S. liquefaciens* cells exposed to Mars-like conditions of 7 mbar, 0 °C, and CO_2_-dominated anoxic atmospheres (called Mars low-PTA conditions). Although not at 7 mbar, one study also explored the transcriptomics of *S. liquefaciens* cells at 50 mbar [13]. Results indicate that *S. liquefaciens* cells are metabolically active and growing down to 7 mbar, even though patterns in the utilization of most amino acids are severely constrained [7]. Furthermore, Schwendner et al. [6] used nano-scale secondary mass spectrometry (nanoSIMS) to demonstrate that the low-temperature threshold for *S. liquefaciens* in a 7 mbar atmosphere is −3 °C; just below the typically tested temperature of 0 °C. In contrast, endospores of seven *Bacillus* spp. were unable to germinate and grow on trypticase soy agar media at 25 mbar or lower under several diverse combinations of simulated Martian conditions [14], but one species—*B. subtilis* 168—could grow in liquid Luria-Broth cultures down to 25 mbar [1].

The primary objective of the current study was to examine the ultrastructure of *S. liquefaciens* cells incubated under simulated Mars low-PTA conditions for 28 days to compare ultrastructural modifications associated with low-pressure, low-temperature, or low pO_2_. Several *Serratia* spp. have been recovered from spacecraft surfaces [15,16,17], and *S. liquefaciens* was used here as a model hypopiezotolerant bacterium. Other *Serratia* type-strains have been shown to be capable of metabolism and growth under simulated Mars low-PTA conditions [4] but were not tested here.

## 2. Methods

### 2.1. Microbial Protocols

Primary cultures of *Serratia liquefaciens* (ATCC 27592) cells were grown on trypticase soy agar (TSA; BD Difco Media, Fisher Scientific, Pittsburgh, PA, USA) at 30 °C for 24 h and harvested for specific experiments described below. A Most Probable Number (MPN) assay [18,19] was used to quantify cell densities titrated in sterile deionized water (SDIW) to yield 2 × 10^6^ cells per ml with a spectrometer. Experimental agar plates were created using sterile loops to spread 25 µL of cells into four quadrants to generate single colonies in the 4th quadrant. Fresh cell suspensions were applied to double-thick TSA plates (30 mL per plate) in deep-dish petri dishes. The deep-dish petri dishes could hold up to 45 mL of media (cat. no. 08-757-28, Fisher Scientific, Pittsburg, PA, USA) and proved to be more effective in maintaining cultures at low pressures than standard petri dishes (25 mL capacity).

### 2.2. Low-Pressure Chambers and Experimental Protocols

Hypobaric conditions were maintained in 4-L polycarbonate desiccators (model 08-642-7, Thermo-Fisher Scientific, Rochester, NY, USA) connected to low-pressure controllers (model PU-842, KNF Neuberger, Trenton, NJ, USA) as described previously [3]. In brief, experimental plates of *S. liquefaciens* cells were placed in the desiccators, four CO_2_-generating anaerobic pouches were inserted around the plates (R681001, AnaeroPack, Remel, Thermo-Fisher Scientific, Rochester, NY, USA), the desiccators were closed, and flushed with pure CO_2_ gas for 2–3 min. All valves on the desiccators were closed, and the units were transferred to microbial incubators capable of holding temperatures down to −10 °C (model 3720A, Thermo-Fisher Scientific, Rochester, NY, USA). The desiccator/KNF controller plumbing was connected, and the internal pressure in the chambers lowered in four steps from 1013 mbar (lab conditions) to 100, 50, 25, and then 7 mbar. Each pressure level was maintained for 30 min before advancing to the next level. The slow depressurization process was required to prevent cracking of the agar surfaces in the plates.

The experimental conditions used here were the following: (1) lab-standard controls were held for 24 h at 1013 mbar, 30 °C, and pO_2_/pN_2_ of 21% and 78%, respectively; (2) low-temperature controls #1 were held 28 d at 1013 mbar, 0 °C, and pO_2_ at 21%; (3) low-temperature controls #2 were held 28 d at 1013 mbar, 0 °C, and pCO_2_ at >99.9%; and (4) low-pressure Mars simulations were held 28 d at 7 mbar, 0 °C, and pCO_2_ at >99.9% (called Mars low-PTA conditions). Previous work by Van Horn et al. [20] demonstrated that the AnaeroPack sachets were capable of quickly scrubbing O_2_ down to <0.1% in small vessels within 1 h. Anaerobic conditions within the desiccators were monitored using anaerobic indicator tablets (model R684002, Remel, Thermo-Fisher Scientific, Rochester, NY, USA). All experimental treatments were incubated in the 4-L desiccators at the indicated temperatures and pressures in separate microbial incubators. Anaerobic sachets were changed every 7 d by venting the desiccators to lab conditions, replacing the sachets and indicator tablets, and repeating the pump-down protocol described above.

### 2.3. Electron Microscopy

Cells of *S. liquefaciens* were maintained for 24 h or 28 d in the four atmospheric treatments as given above. The goal was to compare healthy late log-phase cells in each treatment. The ages of the Earth lab controls (24 h), and the low-temperature and low-pressure treatments (28 d), were based on previous work that established time-steps in which cells retained log-phase growth characteristics [3,5,6,7]. The early work by Schuerger and colleagues [3,5,6,7] grew cells of *S. liquefaciens* on both agar-based media and in liquid cultures for up to 49 days. In both protocols, growth at 0 °C appeared to slow down at approx. 28 d. In preliminary work here, cell growth, colony development, and TEM images of cells incubated at 30 °C suggested that the Earth lab control cells would begin to degrade on TSA at 48 h. Based on the previous work and preliminary experiments here, 24 h and 28 d were selected as the time-steps for generating healthy late log-phase cells for Earth lab controls and the low-temperature and low-pressure treatments, respectively.

Individual pin-prick-sized colonies of *S. liquefaciens* were observable on the Mars low-PTA incubated agar surfaces by 14 d, and grew into 0.5–1.0 mm diameter colonies by 28 d. Colony diameters on all other low-temperature treatments were slightly larger than 1 mm at 28 d. At harvest, cells in individual plates were suspended in 1x phosphate-buffered saline (PBS) and transferred to 1.5 mL microfuge tubes. The PBS buffer was composed of the following and titrated to pH 7.2 with 100 mM KOH or 0.1 N H_2_SO_4_ (all per liter): 8.0 g NaCl, 0.20 g KCl, 1.44 g Na_2_HPO_4_, and 0.24 g KH_2_PO_4_.

Samples were vortexed briefly to disperse cells and mounted on 13-mm diameter 0.40 µm Isopore membrane filters (cat. no. HTTP01300, Merck Millipore, Ltd., Tullagreen-Carrigtwohill, Co., Cork, Ireland) pretreated with an aqueous solution of 0.01% poly-L-lysine (cat no. P8920, Sigma-Aldrich Chemical, Co., St Louis, MO, USA) as a bonding agent. Thimerosal (0.01%; cat. no. T5125, Sigma-Aldrich Chemical, Co., St. Louis, MO, USA) was added to the poly-L-lysine solution as a preservative. The Isopore filters were submerged in the poly-L-lysine solution for 30 min and placed on Whatman No. 4 filter paper to dry. Isopore filters doped with poly-L-lysine were then transferred to the upper surfaces of 1-week-old water agar (WA; 15 g per L, BD Difco Agar, Fisher Scientific, Pittsburg, PA, USA) and approx. 50 µL of *S. liquefaciens* cells were applied to the upper filter surfaces. The PBS buffer would be drawn through the Isopore filters and absorbed by the WA. After 15 min, the Isopore filters were transferred to an electron microscopy fixative.

The following fixation steps were conducted at 24 °C unless otherwise noted. The same protocols were used for scanning electron microscopy (SEM) and transmission electron microscopy (TEM) up to the final ethanol (EtOH) wash (see below). Cells on Isopore filters were fixed in 2% glutaraldehyde (Ted Pella, Inc., Redding, CA, USA) in PBS buffer for 36 h at 4 °C. Cells were warmed to 24 °C over the course of 1 h, rinsed thrice in PBS buffer, post-fixed in similarly buffered 1% osmium tetroxide (Ted Pella) at 24 °C for 2 h, washed thrice in PBS buffer, and then rinsed three times in SDIW. After the final SDIW wash, cells attached to Isopore filters were passed through an ethanol (EtOH) series of 10, 30, 50, 70, 90, and 100%. Cells were rinsed in 100% EtOH three times. Cells were then transferred to the EM-Core labs in the Interdisciplinary Center for Biotechnology Research (ICBR) on the main University of Florida campus in Gainesville, FL, for final processing and imaging.

For SEM, the EM-Core received the cells attached to Isopore filters in 100% EtOH and then critical point dried (CPD) (Autosamdri^®^-815 series A, Tousimis, Rockville, MD, USA) the samples. The CPD dried filters were then mounted on 12 mm carbon conductive adhesive tabs on aluminum stubs (Ted Pella) and sputter-coated with gold-palladium under argon gas (Desk V, Denton Vacuum, Moorestown, NJ, USA). All samples were subsequently imaged with a Hitachi S-4000 or Hitachi SU5000 FE-SEM (Hitachi High Technologies in America, Greenville, SC, USA).

For TEM, the samples were fixed and dehydrated as described above. The EM Core received the cells in 100% EtOH and was further dehydrated with 100% anhydrous acetone. An Araldite/Embed-812 epoxy resin containing Z6040 embedding primer (Electron Microscopy Sciences, Hatfield, PA, USA) was used to infiltrate in increments of 3:1, 1:1, 1:3, followed by 100% Araldite/Embed-812. Resin-infiltrated samples were polymerized at 70 °C for 48 h s. Ultra-thin sections were collected on carbon-coated Formvar mesh grids (FCF100, EMS, Hatfield, PA, USA) post-stained with 2% aqueous uranyl acetate and Reynold’s lead citrate. Sections were examined with a FEI Tecnai G2 Spirit Twin TEM (FEI Corp., Hillsboro, OR, USA), and digital images were acquired with a Gatan UltraScan 2k × 2k camera and Digital Micrograph software (version 1.93.1362, Gatan Inc., Pleasanton, CA, USA). Cell-wall thicknesses (def., by Hobot [21] and Madigan et al. [22]) for all treatments were measured using the internal biometric analysis program within the FEI Tecani TEM scope or with Figi version v1.54i [23].

### 2.4. Statistics

The biometric thicknesses of cell walls in the divergent *S. liquefaciens* atmospheric treatments were collected from TEM micrographs. Cell-wall thicknesses were measured at two locations on opposite sides of individual cells in separate TEM images. The two measurements were averaged, and the observations (*N* in Table 1) were analyzed with the Statistical Analysis System (SAS), version 9.4 (SAS Institute, Inc., Cary, NC, USA). Untransformed data for each atmospheric treatment were analyzed with PROC GLM for ANOVA and LSmeans tests (*p* ≤ 0.05; Table 1; Table S1).

## 3. Results

Growth of *S. liquefaciens* cultures at 7 mbar was facilitated by using double-thick agar pours in deep-dish petri plates. If the initial pump-downs of the desiccators were stepped from 100 to 7 mbar, as described above, the agar surfaces would not bubble or split. Low-pressure assays with semi-solid agar media must be permitted to slowly outgas dissolved lab gases (i.e., pO_2_ and pN_2_) to prevent agar defects. Colonies of *S. liquefaciens* under all conditions maintained at 0 °C would appear between 10 and 14 d as pin-prick-sized colonies (def., visually visible but not easily measurable with hand-held micrometers). After 28 d, colonies at 7 mbar were approx. 0.5 to 1.0 mm in diameter. The colonies in all other low-temperature treatments (i.e., incubated at 0 °C) were similar in color, texture, and morphology, except for slight differences in size.

**Figure 1:**
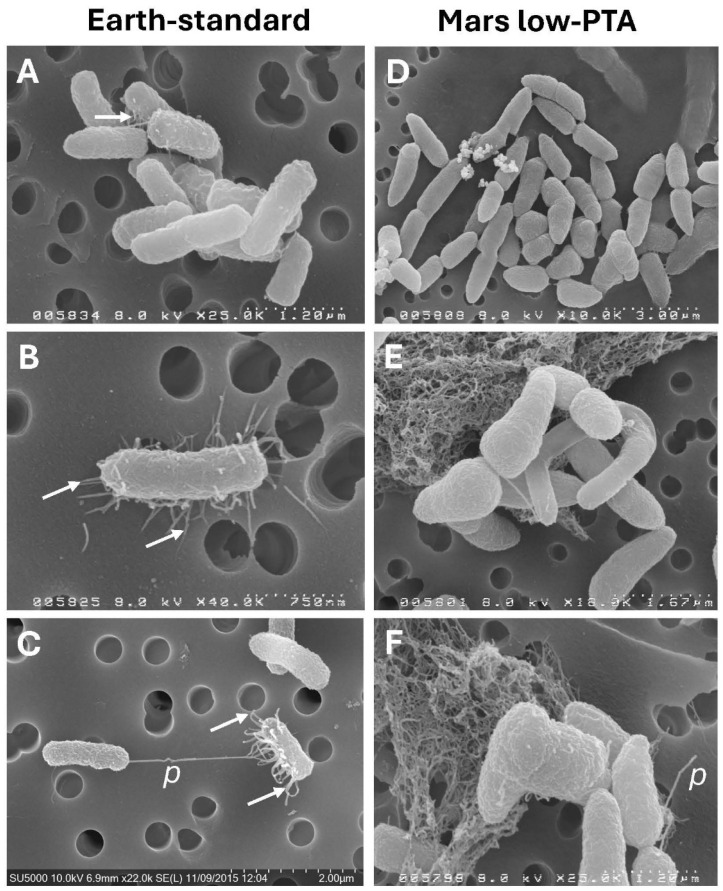
Scanning electron micrographs of *Serratia liquefaciens* cells grown under Earth standard conditions and Mars low-PTA conditions for 24 h or 28 d, respectively. (**A**) Earth standard conditions were 1013 mbar, 30 °C, and 21%/78% pO_2_/pN_2_ atmospheres. Cells were rod-shaped, measuring approx. 1.25 µm long by 0.5 µm wide and exhibited fimbriae [arrows] (**B**) and nanotubes [*p*] (**C**) in approx. 10% of the cells. Cells incubated under low-PTA conditions of 7 mbar, 0 °C, and CO_2_-enriched anoxic atmospheres were longer than the controls and exhibited tapered ends of progeny cells (**D**,**E**), some right-angled divisions (**F**), and fimbriae and nanotubes were generally lacking. The debris in Figure 1E,F was likely extracellular matrix from the *S. liquefaciens* cultures. The extracellular matrix was present in all treatments, but only shown here due to the proximity to morphologically chosen cells in Figure 1E,F. Scale bars are present in the lower right corners of all images.

Cells of *S. liquefaciens* grown under the lab-standard conditions of 1013 mbar, 30 °C, and an Earth-normal pO_2_/pN_2_ atmosphere were approx. 1 to 1.25 µm in length and 0.5 µm in width (Figure 1A). Distorted cells were lacking. Fimbriae (Figure 1B) and nanotubes (Figure 1C; syn. pili) were common in lab-standard control cultures. Subtle cell divisional plate indentations (Figure 1A) were observed in elongated cells that indicated normal cell division was underway. In contrast, cells of *S. liquefaciens* grown in Mars’ low-PTA conditions of 7 mbar, 0 °C, and CO_2_-dominated anoxic atmospheres exhibited numerous defects that diverged from the Earth controls. Most notably, bound daughter-cell doublets were very common, in which indentations of divisional plates were pronounced (i.e., deeper as compared to Earth lab-standard controls) and the distal ends of the daughter cells dramatically tapered (Figure 1D–F). In addition, many of the Mars low-PTA-grown cells exhibited right-angle divisions (Figure 1D,F) or elongated and spiral morphologies. The frequencies of fimbriae and nanotubes were subdued in the Mars low-PTA conditions compared to the lab-standard Earth controls. Extracellular matrix was observed in all treatments, but is only shown here in Figure 1E,F.

**Figure 2:**
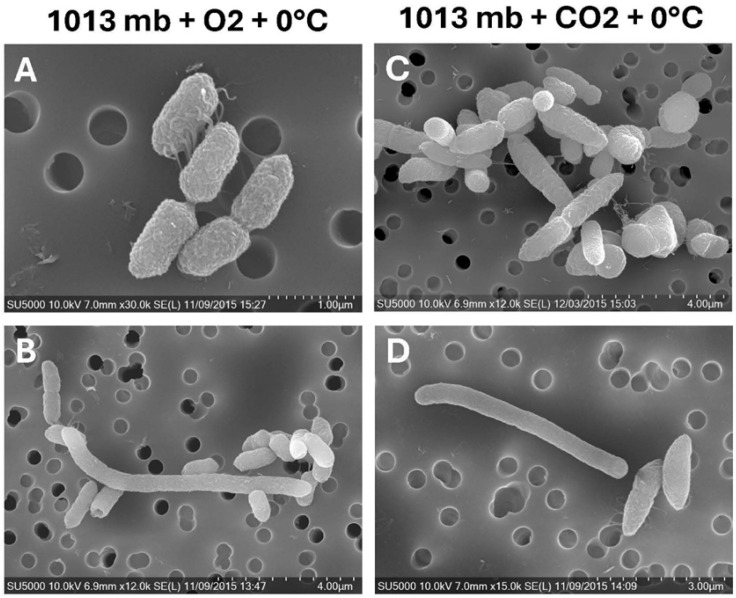
Scanning electron micrographs of *Serratia liquefaciens* cells grown under two Earth low-temperature controls for 28 d. (**A**,**B**) Cells grown under low-temperature conditions of 0 °C—but normal pressure (1013 mbar) and lab-standard atmospheres of pO_2_/pN_2_—were similar to lab-standard controls except for slightly tapered ends of progeny cells and periodically remaining in long chains of cells that failed to divide normally. (**C**,**D**) Cells grown at 0 °C and under normal pressure–but with CO_2_-enriched anoxic gas composition–exhibited slightly increased rates of tapered distal ends of progeny cells, and more cells were present as doublets in which cells failed to properly divide. Scale bars are present in the lower right corners of all images.

Two additional Earth low-temperature controls were included in the assays to measure the effects of only temperature (low-temperature control #1) or low temperature + gas composition (low-temperature control #2) on cell morphologies. Low-temperature control #1 (i.e., 1013 mbar, 0 °C, and pO_2_ 21%) with *S. liquefaciens* cells exhibited nearly normal morphologies with only slightly tapered distal ends (Figure 2A) and infrequent elongated cells (Figure 2B). Adding the second stress factor of high pCO_2_ to low temperature (0 °C), the Earth control #2 cells exhibited morphologies more similar to the Mars low-PTA conditions. Specifically, cells exhibited slowed division (i.e., more paired daughter cells), deeper divisional plate indentations, more severe tapered distal ends, and right-angled daughter cells (Figure 2C,D). Furthermore, fimbriae and nanotubes were subdued in the low-temperature controls #1 and #2 when compared to the lab-standard controls at normal pressure, temperature, and gas composition.

Based on the nomenclature of Hobot [21] and Madigan et al. [22], the structures that surround Gram [−] bacteria, like *S. liquefaciens*, can be defined as cell walls that are composed of (i) an outer membrane, and (ii) a thin periplasmic gel composed of cross-linked peptidoglycans. The cells walls of Gram [+] bacteria lack the outer membranes, have structures called the S-layers above thicker and more chemically complex internal structures composed of peptidoglycans, anionic polymers, and lipoteichoic acid. During TEM imaging of the cell walls for *S. liquefaciens*, the complex of (i) the outer membranes and (ii) the periplasmic gel layers were measured for cells exposed to each treatment. The inner cytoplasmic membranes were very thin in all atmospheric treatments and were not included in the measurements of cell-wall thicknesses.

**Figure 3:**
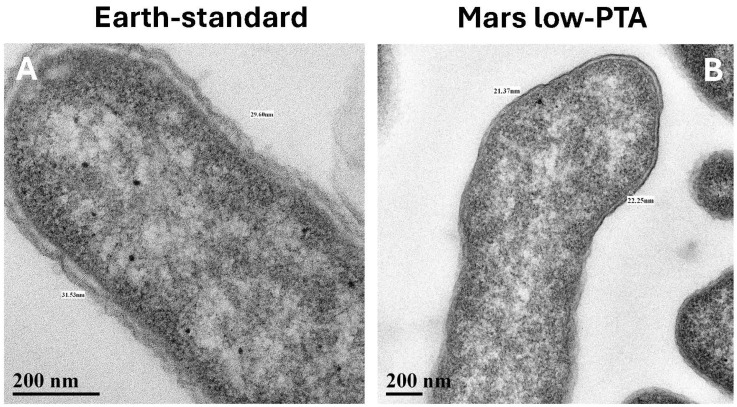
Transmission electron micrographs of *Serratia liquefaciens* cell walls grown under lab-standard (24 h) and Mars low-PTA conditions (28 d) (see text). (**A**) Cell walls of *S. liquefaciens* cells grown under lab-standard conditions measured on average 29.4 µm thick as measured from the inside of the periplasmic layer and ending at the outside of the outer membrane (i.e., similar to Gram-negative cell walls described by Hobot [21]). (**B**) In contrast, cell walls were significantly thinner in *S. liquefaciens* when grown under Mars low-PTA conditions and measured approx. 24.3 µm. The cell-wall thicknesses of the other Earth controls incubated for 28 d at low temperatures fell between the two extremes given above (see Table 1). Scale bars are present in the lower left corners of all images. [Magnifications: (**A**) = 1600×; (**B**) = 1100×].

**Figure 4:**
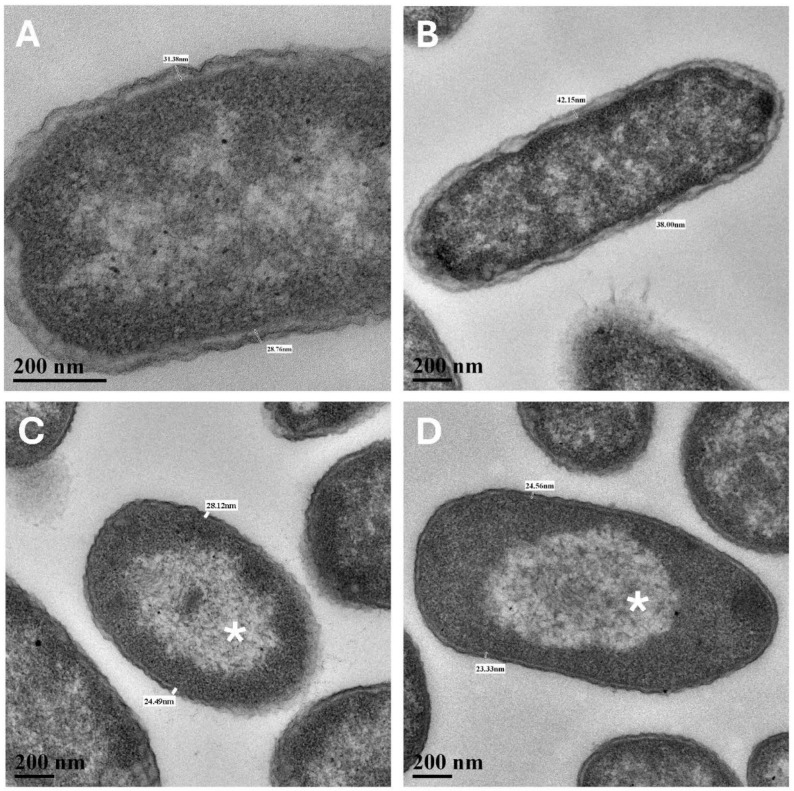
Nucleoids in *Serratia liquefaciens* cells grown under Earth standard (24 h) and Mars low-PTA (28 d) conditions. Cells grown under lab-standard conditions (**A**) and under pO_2_ of 21% at 0 °C (**B**) (see text) did not exhibit well-defined nucleoids, but instead, cells were composed of diffuse structures, including ribosomes, DNA, and lipoprotein bodies. Approximately 10% of cells grown under Mars low-PTA (**C**) or under pCO_2_ at 0 °C (**D**) conditions exhibited diffuse small nucleoids (asterisks) and a denser packing of ribosomes and cytosolic structures on the periphery of the cells. Scale bars are present in the lower left corners of all images. [Magnifications: (**A**) = 1600×; (**B**–**D**) = 1100×].

**Figure 5:**
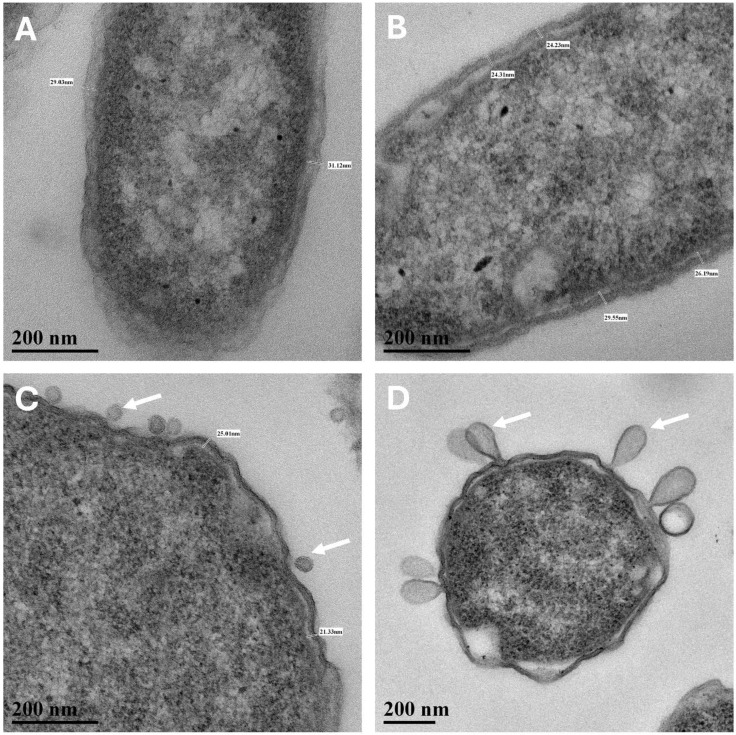
Outer membrane vesicles (OMV; arrows) were not observed in *Serratia liquefaciens* cells grown under lab-standard conditions for 24 h at 30 °C (**A**) or at 0 °C, 1013 mb, and pO_2_ (21%) (**B**). In contrast, OMV were observed on approx. 10% of cells grown under CO_2_-enriched atmospheres under low-PTA conditions (28 d) (**C**) or under CO_2_-enriched atmospheres at 0 °C and 1013 mbar (28 d) (**D**). Cell-wall biometrics were not measured for Figure 5D. Scale bars are present in the lower left corners of all images. [Magnifications: (**A**–**C**) = 1600×; (**D**) = 1100×].

In TEM cross- and longitudinal-sections, cell walls of the Mars low-PTA-grown cells were noticeably thinner than the Earth lab-standard controls (Figure 3A and Table 1). The average cell-wall thickness of Mars low-PTA-grown cells was 24.3 nm compared to 29.4 nm for the lab-standard controls, a 17% decrease. Cell walls of *S. liquefaciens* cells grown under the Earth low-temperature control #1 conditions were not significantly different from the lab-standard controls, suggesting that low temperature alone did not induce a thinning of the cell walls. In contrast, when cells were incubated under the Earth low-temperature control #2 conditions that combined low-temperature (0 °C) with a high pCO_2_ atmosphere (>99.9%), cell walls were similar to, but significantly thicker than, the low-PTA grown cells (Table 1). Second, TEM imaging revealed that small well-defined nucleoids (approx. 10% of cells) were common in the Mars low-PTA conditions (Figure 4C) and in the low-temperature controls #2 (Figure 4D) but rare in the Earth lab-standard (Figure 4A) and pO_2_ (Figure 4B) controls. In general, the nucleoids for the Earth controls were diffusely distributed throughout the cells. And third, *S. liquefaciens* cells incubated under low-PTA conditions at 7 mbar or low-temperature control #2 conditions at 1013 mbar, 0 °C, and high-CO_2_ atmosphere exhibited frequent evaginations of the outer cell-wall membrane (Figure 5) called outer membrane vesicles (OMV) [24,25]. In general, OMVs are produced by Gram-negative bacteria in response to environmental stressors, host-pathogen interactions, and contact with other environmental microbiomes [24,25]. The small and large evaginations depicted in Figure 5B and 5C, respectively, were not observed in Earth lab-standard controls or in the low-temperature control #1 cells.

## 4. Discussion

Characterizing the ultrastructure of microbial cells grown under hypobaric conditions may offer insights into the cellular mechanisms that allow adaptations to extreme conditions in Earth’s stratosphere and on the surface of Mars. The results presented here are the first report of ultrastructural alterations in the eubacterium, *Serratia liquefaciens*, caused by synergistic interactions among the three factors of low pressure, low temperature, and CO_2_-dominated anoxic conditions. The ultrastructure of control cells incubated under lab-standard conditions of 1013 mbar, 30 °C, and lab air of pO_2_/pN_2_ (21% and 78%, respectively) was similar to the descriptions of Gram-negative bacteria given in a recent review by [21].

Overall, results indicated that as each environmental parameter was applied to populations of *S. liquefaciens* cells, daughter cells failed to readily separate along divisional plates, distal ends of daughter cells became constricted, cell walls thinned, right-angled or spiral daughter cells formed, cellular nucleoids shrank, and outer membrane vesicles increased in size and number. The more stress factors applied, the more these ultrastructural alterations were observed. The evidence suggests that the alterations were most likely caused by decreasing pO_2_ in the hypobaric chambers. A direct low-pressure effect was not overtly identified.

Similar ultrastructural changes have been noted in other taxa of eubacteria and archaea under extreme conditions of low temperature and low pressure. First, the frequency of cojoined daughter cells and tapered distal ends of daughter cells both increased for *S. liquefaciens* cells grown at 4 °C compared to 37 °C; the alterations appeared to be caused solely by incubation at low temperatures [26]. Second, cell morphology, shape, and size were altered and constrained in cells of the archaeon *Haloferax volcanii* incubated at 24 mbar; the changes were caused by low-pressure effects alone because both the control cells (1013 mbar) and experimental cells (24 mbar) were grown at 21 °C [27]. Third, incubation of *Bacillus subtilis* cells at 15 °C compared to controls at 37 °C yielded spiral cell clusters composed of many daughter cells in which division was inhibited [28]. Similar spiral, curved, or constricted malformations of cell aggregates were observed in the archaeon, *Methanobacterium thermoautotrophicum*, when cells were grown at temperatures lower (45 °C) or higher (75 °C) than the optimum range (65 °C) [29]. And fourth, constricted nucleoids observed here under Mars-like low-PTA conditions have been reported for vegetative cells of the eubacteria *Escherichia coli* and *B. subtilis* grown on diverse nutrient regimes [30]; and at low temperatures (−10 °C compared to 22 °C) for cells of the permafrost bacterium, *Psychrobacter cryopegella* [31]; now called *P. cryohalolentis*).

However, direct metabolic and ultrastructure links for *S. liquefaciens* cells grown under low-PTA conditions are lacking. A few insights may be possible by comparing the current results with three recently published papers on the metabolism and transcriptomics of *S. liquefaciens* incubated under low-PTA conditions at 7 mbar. First, carbon source utilization by *S. liquefaciens* under low-PTA conditions changed dramatically, with cells unable to utilize most amino acids in 95-well Biolog microbial assay plates compared to controls [7]. In contrast, most organic sugars in the Biolog assay plates were able to support cellular metabolism and growth. Second, the addition of several anaerobic electron acceptors for redox couples in 125 eubacteria—including *S. liquefaciens*—failed to boost growth rates under low-PTA conditions, suggesting that the addition of vitamins, nitrate, sulfate, or iron does not enhance existing metabolic pathways supporting bacterial growth at low pressures [5].

And third, in a recent transcriptomics study of *S. liquefaciens* cells incubated under low-PTA conditions versus lab-standard conditions identical to those described here [13], 193 genes were up-regulated in the low-PTA exposed cells that included genes (a) encoding ABC and PTS transporters, (b) involved in translation (e.g., ribosomes biogenesis, tRNA biosynthesis, and aminoacyl-tRNAs), (c) DNA repair and recombination, and (d) non-coding RNAs. Conversely, 300 genes were down-regulated in the low-PTA incubated cells that were part of the following pathways: (i) flagellar and motility proteins, (ii) genes involved in phenylalanine metabolism, (iii) transcription factors, and (iv) two-component systems. In general, the indicated up- and down-regulation of the genes listed above suggests that signaling and transport processes across cell membranes might have been severely impacted at low pressures. This conclusion is supported by a report on alterations in membrane fluidity in vegetative cells of *B. subtilis* grown under decreasing pressures down to 25 mbar in which saturated fatty acids (FAs) increased and unsaturated FAs decreased in cells grown at decreasing pressures, resulting in reduced membrane fluidity at low pressures [1]. Saturated FAs in bacterial cell membranes tend to lower membrane fluidity and function. In contrast, unsaturated FAs possess increased numbers of carbon-carbon double bonds, which can create conformational changes that inhibit tight packing and increase membrane fluidity.

Two interesting and possibly confounding factors not studied here are (1) the adaptability of *S. liquefaciens* cells exposed to Mars low-PTA conditions and returned to Earth lab standard conditions, and (2) the effects of long-term maintenance of cells on TSA with regard to nutrient deprivation. First, Schuerger et al. [3] observed that cells of *S. liquefaciens* exposed to Mars low-PTA conditions for up to 35 d and then returned to Earth-normal lab conditions exhibited normal growth and development. In the current study, we did not repeat this procedural step because the cells were harvested for SEM and TEM fixation. However, we believe that the cells would have resumed normal growth had they been transferred back to Earth-normal lab conditions. Thus, adaptations to low-PTA conditions are not permanent.

And second, long-term storage of *S. liquefaciens* cells on TSA for 28 d in treatments incubated at 0 °C may have been impacted by nutrient deprivation of the medium. However, in earlier studies [3,4,5,6,7], cells of *S. liquefaciens* were exposed to both agar-based and liquid media for up to 49 d without exhibiting unusual, slowed, or degraded growth. In fact, colonies on TSA under low-PTA and low-temperature conditions (i.e., 0 °C) in the current study typically required up to 14 d to be visually observable, suggesting a long lag phase at 0 °C. Furthermore, Schwendner et al. [6] demonstrated that the minimum temperature at which *S. liquefaciens* ATCC 27592 could grow was −3 °C, very close to the incubation temperatures used here. The goal of the current work was to examine healthy late log-phase cells incubated at Earth-normal lab conditions at 30 °C for 24 h to healthy cells in late log-phase growth incubated for 28 d under low-PTA (or other 0 °C) conditions. In all treatments described here—and consistent with earlier studies [3,4,5,6,7]—cells appeared healthy with intact membranes and cell-wall structures even if incubated for 28 d at 0 °C. However, nutrient deprivation in growth media should be considered in future work.

Based on the discussion above, the ultrastructural changes reported here under low-PTA conditions might be linked to inhibition of (a) membrane transport of bioessential nutrients—especially amino acids, (b) DNA repair and recombination, (c) transcription and translation, (d) signal transduction pathways, (e) membrane fluidity at low pressure, or (f) nutrient deprivation. Inhibition of these pathways would have placed significant resource constraints on *S. liquefaciens* cells that interfered with cell division, cell-wall thickness, and cellular organization (e.g., constriction of daughter-cell distal ends and the structure of nucleoids). New research is suggested to probe direct links between the transcriptomes, metabolomes, and ultrastructure in hypopiezotolerant bacteria and archaea incubated under low-pressure conditions. Characterizing the links among these three topics may provide new insights into how hypopiezotolerant bacteria and archaea are capable of metabolism and growth under low-PTA conditions found in the Earth’s stratosphere and on the surface of Mars.

## 5. Conclusions

Recent studies have identified up to 30 bacteria [4] and one archaeon (i.e., *Methanosarcina barkeri* [8]) capable of metabolism and growth under low pressure. The current study is the first to examine ultrastructure changes in the hypopiezotolerant bacterium, *Serratia liquefaciens*, grown under low-PTA conditions. However, the mechanisms by which bacteria or archaeal species can tolerate and thrive under low-PTA conditions are unknown. Interestingly, environmental conditions of pressure, temperature, and low pO_2_ in the upper stratosphere on Earth (i.e., between 42 and 50 km) are similar to those found on the surface or shallow subsurface of Mars. Thus, characterizing the cellular processes that confer tolerance to Mars low-PTA conditions in terrestrial microorganisms might provide insights into (i) how the terrestrial aerobiology can survive and thrive in Earth’s stratosphere, (ii) how spacecraft microorganisms might survive and proliferate on Mars, and (iii) how to search for an extant microbiota in the shallow subsurface of the red planet.

## Figures and Tables

**Table 1:** Cell-wall thicknesses for *Serratia liquefaciens* cells under Earth control and Mars low-PTA conditions.

Treatments	Cell-Wall Thickness (nm)	*N* Observations
Lab-standard control (1013 mbar + 30 °C + pO_2_)	29.4 * a	30
Low-temperature Con-1 (1013 mbar + 0 °C + pO_2_)	28.7 a	34
Low-temperature Con-2 (1013 mbar + 0 °C + pCO_2_)	26.6 b	42
Mars (7 mbar + 0 °C + pCO_2_)	24.3 c	31

* Divergent letters indicate significant differences among the means based on an ANOVA and a least-squares mean separation test (*p* ≤ 0.05). Data were not transformed prior to analysis.

## Data Availability

The original contributions presented in this study are included in the article/Supplementary Material. Further inquiries can be directed to the corresponding author.

## Supplementary Materials

The following supporting information can be downloaded at: https://www.mdpi.com/article/10.3390/microorganisms13112466/s1, Table S1: TEM cell wall data.
